# Tumor suppressor microRNA-34a inhibits cell proliferation by targeting Notch1 in renal cell carcinoma

**DOI:** 10.3892/ol.2014.1931

**Published:** 2014-03-04

**Authors:** CHANGCUN ZHANG, REN MO, BINDE YIN, LIBIN ZHOU, YONGCHAO LIU, JIE FAN

**Affiliations:** Department of Urology, Shanghai First People’s Hospital, School of Medicine, Shanghai Jiaotong University, Shanghai 200080, P.R. China

**Keywords:** notch1, microRNA-34a, renal cell carcinoma, target oncogenes, cell proliferation, cell cycle

## Abstract

MicroRNA-34a (miR-34a) is a tumor suppressive microRNA, which induces G1 arrest, apoptosis and senescence by repressing the expression of multiple oncogenes. This study aimed to investigate the biological function and molecular mechanisms of miR-34a in human renal cell carcinoma (RCC) cells. Quantitative polymerase chain reaction (qPCR) revealed that miR-34a expression was significantly downregulated in eight of the 10 (80%) RCC tissues compared with adjacent normal tissues. In RCC cell lines, several other target genes of miR-34a were dysregulated at the mRNA level when the expression of miR-34a was elevated. In addition, western blot analysis and qPCR revealed that forced expression of miR-34a downregulated the expression of Notch1 at the protein and mRNA level. The Cell Counting Kit-8 identified that transient forced expression of miR-34a inhibited cell growth and resulted in cell cycle arrest, which was evaluated by flow cytometry. Our data demonstrated that miR-34a inhibits cell proliferation by downregulating Notch1 in RCC cell lines.

## Introduction

MicroRNAs (miRNAs), which are a conserved class of short, non-coding, endogenous RNAs comprising ~22 nucleotides, are involved in post-transcriptional gene regulation by inhibiting translation or cleaving specific target mRNAs (1). It has been indicated that miRNAs play important regulatory roles in various biological processes, such as cell proliferation, developmental timing, apoptosis, metabolism and carcinogenesis (2). miRNAs have been classified as oncogenes or tumor suppressor genes according to their function in carcinogenesis and ectopic expression in tumors (3). For example, miR-27a is overexpressed and functions as an oncogene in the development of renal cell carcinoma (RCC) (4), and Let-7a may inhibit cell growth by targeting c-myc in RCC (5).

MicroRNA-34a (miR-34a) is a direct target of p53 (6) and is commonly downregulated in several types of cancer. For example, miR-34a was downregulated in 10 out of 10 prostate cancer tissues (7). Gallardo *et al* (8) demonstrated that miR-34a expression was significantly reduced in non-small-cell lung cancer compared with normal tissues. The ectopic expression of miR-34a may induce G1 arrest (9–11), apoptosis and senescence in tumors (6,12,13). Moreover, miR-34a directly regulates numerous target genes that mediate its diverse antitumor effects, and several target genes of miR-34a have been reported, including Notch1 (14,15), cyclin-dependent kinase 6 (CDK6) (11), E2F transcription factor 3 (E2F3) (12), cyclin E2 (CCNE2) (16) and silent information regulator 1 (SIRT1) (17).

The Notch signaling pathway is highly conserved and regulates the biological processes involved in cell fate specification, differentiation, proliferation, apoptosis, adhesion, migration and angiogenesis (18). Abnormal Notch signaling has been found in the tumorigenesis of cancers (19–21) and ectopic expression of Notch signaling is also involved in the carcinogenesis of RCC (22,23). Previous studies have shown that miR-34a regulates the expression of Notch1 in a number of human cancers (14,15). Thus, this study hypothesized that there is a significant correlation between antineoplastic miR-34a and oncogenic Notch1 in the development of RCC. This study aimed to investigate the biological function and molecular mechanisms of miR-34a in human renal cell carcinoma cells.

## Materials and methods

### Tissue samples

Patient-matched RCC (10 pairs) and normal renal tissues were obtained from patients who underwent radical nephrectomy at the Department of Urology, Shanghai First People’s Hospital, School of Medicine, Shanghai Jiaotong University (Shanghai, China) between 2006 and 2010. The normal renal tissues were obtained from a distance of ≥5 cm from the tumor tissues. Paired tissue specimens (n=10) were identified as clear cell RCCs with four, five and one specimen classified as stages I, II and III, respectively, according to clinical tumor-node-metastasis staging (American Joint Committee on Cancer); whereas one, seven and two RCCs were classified as grades I, II and III, respectively, according to their differentiation status. The clinical and pathological characteristics of the renal samples are presented in Table I. All the specimens were snap-frozen in liquid nitrogen immediately and stored at −80°C following surgery, until RNA extraction. The histological diagnosis was confirmed by examining hematoxylin and eosin-stained original sections simultaneously by two pathologists. The The Research and Ethics Committees of Shanghai First People’s Hospital (Shanghai, China) approved the study, and patient consent was obtained prior to tissue collection.

### Cell culture

The 786-O and Caki-1 human RCC cell lines were obtained from the Cell Resource Center of Shanghai Institutes for Biological Sciences, Chinese Academy of Sciences (Shanghai, China). The 786-O cells were cultured in RPMI-1640 medium (Gibco-BRL, Rockville, MD, USA) supplemented with 10% fetal bovine serum (FBS; Gibco-BRL) and 100 U/ml penicillin/streptomycin (Invitrogen Life Technologies, Carlsbad, CA, USA). The Caki-1 cells were cultured in McCoy’s 5A medium (Gibco-BRL) supplemented with 10% FBS and 100 U/ml penicillin/streptomycin. The cells were cultured in a humidified incubator in an atmosphere of 5% CO_2_ at 37°C.

### Cell transfection

The 786-O and Caki-1 cells were seeded at 1×10^5^ cells per well in 6-well plates and transfected with 50 nM of miR-34a mimics or the negative control (GenePharma, Co., Ltd., Shanghai, China) using Lipofectamine^®^ RNAiMAX (Invitrogen Life Technologies) according to the manufacturer’s instructions. The cells were then harvested 48 h after transfection.

### RNA extraction

Total RNA from cultured cells and tissues was extracted using the TRIzol reagent (Invitrogen Life Technologies) according to the manufacturer’s instructions. RNA quantity and quality were determined by spectrophotometry at 260 nm (SmartSpec Plus, Bio-Rad, Hercules, CA, USA) and agarose gel electrophoresis (SantaiBio, Shanghai, China).

### Quantitative polymerase chain reaction (qPCR)

Reverse transcription for miRNAs was performed using the TaqMan MicroRNA Reverse Transcription kit (Applied Biosystems, Foster City, CA, USA). TaqMan MicroRNA expression assays (Applied Biosystems) were used to provide specific primers for the reverse transcription and quantitation of mature miR-34a and RNU6B. Thermal cycling conditions for qPCR were as follows: 95°C for 10 min, followed by 40 cycles of 95°C for 15 sec and 60°C for 60 sec. RNU6B expression was used as an internal control for miR-34a expression. In order to detect mRNA, 500 ng of RNA was reverse-transcribed using a PrimeScript Reverse Transcriptase reagent kit (Takara, Dalian, China) according to the manufacturer’s instructions, and was amplified by qPCR using specific primers (Table II). Glyceraldehyde-3-phosphate-dehydrogenase (GAPDH) was used as an internal control to normalize for differences in the input RNA. The PCR conditions were as follows: One cycle at 95°C for 1 min and 34 cycles at 94°C for 10 sec, 60°C for 30 sec and 72°C for 15 sec. All the measurements were performed in triplicate. Amplification was analyzed using the ΔΔCt method (24).

### Cell proliferation analysis by Cell Counting Kit-8 (CCK-8)

The cells were seeded into 96-well plates at 1,000 cells per well 24 h after transfection. The effects of miR-34a on cell proliferation were detected 0, 24 and 48 h after seeding using CCK-8 (Dojindo Molecular Technologies, Inc., Kumamoto, Japan) according to the manufacturer’s instructions. Each assay was performed in five replicates on three independent experiments.

### Cell cycle analysis by flow cytometry

After transfection (48 h) and prior to cell counting, the cells were collected, washed with cold phosphate-buffered saline (PBS), fixed in cold 70% ethanol in PBS for at least 24 h and labeled with propidium iodide. After staining, cells were counted on FACSCalibur using CellQuest Pro software (BD Biosciences, Franklin Lakes, NJ, USA). The cell cycle fractions were analyzed using ModFit software, version 3.0 (BD Biosciences).

### Western blot analysis

Total protein was extracted from the cells using whole cell lysates (Beyotime, Jiangsu, China). The protein concentrations of individual samples were assessed using a standard bicinchoninic acid assay (Beyotime). For each sample, 60 μg of protein was separated on 10% SDS-PAGE gel (Bio-Rad), transferred onto a polyvinylidene difluoride membrane (catalogue number: 3010040001; Roche Applied Science, Mannheim, Germany)and blocked with 5% skimmed milk and 0.1% Tris-buffered saline-Tween 20 (TBST) at room temperature for 1.5 h. The membranes were washed in TBST three times and incubated overnight at 4°C with monoclonal rabbit anti-Notch1 antibody (1/1,000 dilution) and monoclonal rabbit anti-GAPDH antibody (1/8,000 dilution). The primary antibodies recognize human protein and were purchased from Cell Signaling Technology, Inc. (Beverly, MA, USA). The membranes were then washed with 1X TBST (Jiuzhou Tech, Beijing, China) and incubated with anti-rabbit IgG horseradish peroxidase-conjugated secondary antibody (Cell Signaling Technology, Inc.). The protein expression was evaluated using chemiluminescence and exposure to Kodak film.

### Statistical analysis

Data are presented as the means ± standard deviation from at least three separate experiments and significance was analyzed using the Student’s t-test. All the analyses were performed using SPSS software, version 17.0 (SPSS Inc., Chicago, IL, USA). P<0.05 is considered to indicate a statistically significant difference.

## Results

### miR-34a expression is downregulated in renal tumors

**Figure 1:**
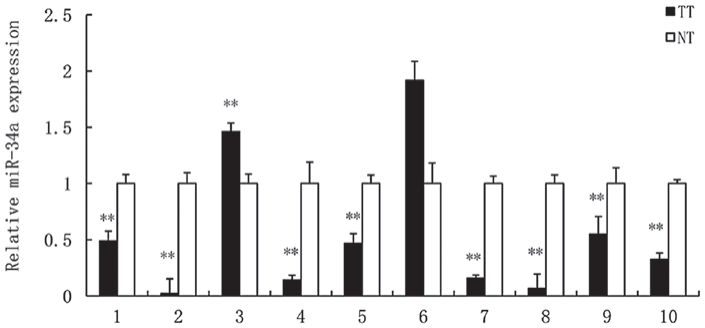
Relative expression of miR-34a in 10 pairs of patient-matched RCCs and normal renal tissues examined by quantitative polymerase chain reaction. Data shows the expression levels of miR-34a in RCC relative to the normal renal samples. The mean and standard deviation of expression levels relative to RNU6B expression levels are shown and are normalized to the expression in the normal tissue of each matched pair. ^*^P<0.05 and ^**^P<0.001, compared with paired NT. All the experiments were performed three times independently. miR-34a, microRNA-34a; RCC, renal cell carcinoma; TT, tumor tissue; NT, normal tissue.

To investigate the underlying mechanisms of miR-34a in the development of RCC, the expression levels of miR-34a in human RCC tissues and adjacent normal tissues were examined by qPCR. miR-34a was significantly downregulated in eight of the 10 (80%) human RCC tissues compared with the adjacent normal tissues (Fig. 1). These findings suggest that the downregulation of miR-34a may be involved in human RCC carcinogenesis.

### Functional effects of miR-34a on proliferation and cell cycle progression in RCC cell lines

**Figure 2:**
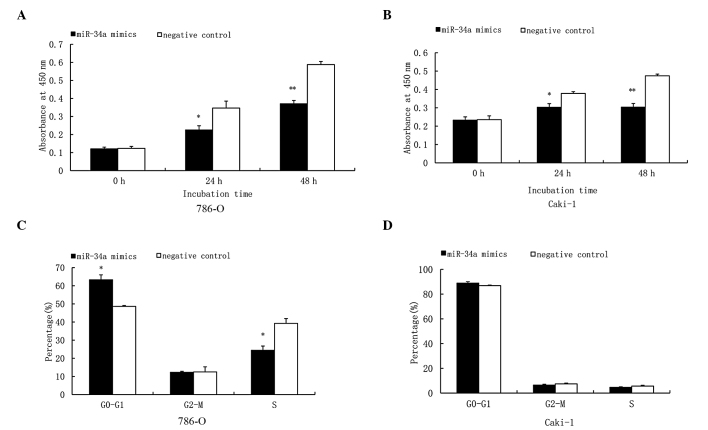
Functional effects of miR-34a on growth and cell cycle distribution in RCC cell lines. (A and B) Suppressive effects of miR-34a on the growth of 786-O (A) and Caki-1 (B) cells. After transfection (24 h), cells were seeded into 96-well plates at a density of 1,000 cells per well. The effect of miR-34a on cell proliferation was detected at 0, 24 and 48 h after seeding using Cell Counting Kit-8. (C and D) The cell cycle distribution of 786-O (C) and Caki-1 (D) cells transfected with miR-34a mimics compared with the negative control showed that cell cycle distribution varied between the two RCC cell lines. Data are expressed as the means ± SD. ^*^P<0.05 and ^**^P<0.01, compared with the negative control. All the experiments were performed three times independently. miR-34a, microRNA-34a; RCC, renal cell carcinoma.

The effects of miR-34a on cell proliferation in two RCC cell lines was investigated. To validate the antiproliferative function of miR-34a in RCCs, the RCC cell lines were transfected with miR-34a mimics and the negative control. The proliferation rate of each RCC cell line 24 h after transfection and over the following three days was examined using CCK-8. Our findings suggested that the overexpression of miR-34a significantly inhibited the cell proliferation rate compared with the negative control in the two RCC cell lines (Fig. 2A and B). Flow cytometry examined the functional effects of miR-34a on cell cycle progression. Different alterations in the cell cycle were observed in the two RCC cell lines (Fig. 2C and D). A significant decrease in the percentage of cells in S phase and a significant arrest in the G0–G1 phase was observed in the 786-O cells (Fig. 2C); whereas no significant decrease in the percentage of cells in S phase was observed in Caki-1 cells, but a significant arrest in the G0–G1 phase was identified (Fig. 2D).

### Relative mRNA expression levels of putative target genes of miR-34a in RCC cell lines

**Figure 3:**
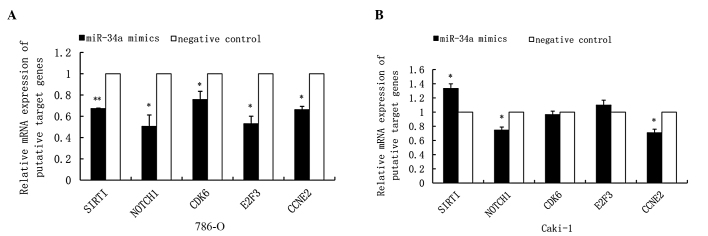
Relative mRNA expression of miR-34a on putative target genes in (A) 786-O and (B) Caki-1 cells transfected with miR-34a mimics or the negative control. Data are expressed as the means ± SD. ^*^P<0.05 and ^**^P<0.01, compared with the negative control. All the experiments were performed three times independently. miR-34a, microRNA-34a; SIRT1, silent information regulator 1; CDK6, cyclin-dependent kinase 6; E2F3, E2F transcription factor 3; CCNE2, cyclin E2.

The molecular mechanisms of miR-34a in the development of RCC were further investigated. miR-34a exerts its diverse inhibitory effects by regulating a number of target genes. According to the bioinformatic analysis on TargetScan (http://www.targetscan.org/), SIRT1, Notch1, CDK6, E2F3 and CCNE2 are putative target genes of miR-34a and have been found in different tumors. In the present study, the mRNA expression levels of these genes following transfection were examined by qPCR. Our data showed that the mRNA expression levels of SIRT1, Notch1, CDK6, E2F3 and CCNE2 in the 786-O cell line were significantly reduced in the miR-34a mimics group compared with the negative control group (Fig. 3A). In the Caki-1 cell line, Notch1 and CCNE2 were downregulated and miR-34a exerted almost no significant effects on the mRNA levels of SIRT1, CDK6 and E2F3 (Fig. 3B).

### Notch1 is downregulated by miR-34a in the RCC cell lines

**Figure 4:**
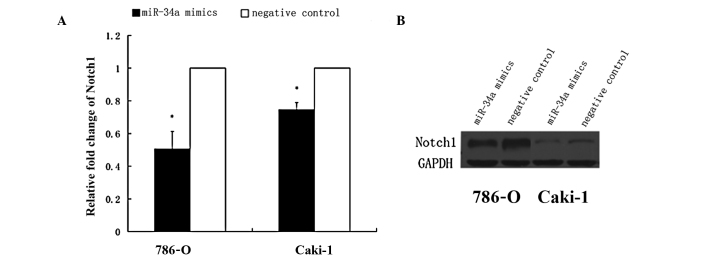
Downregulation of Notch1 by miR-34a in the RCC cell lines. (A) Quantitative polymerase chain reaction analysis of Notch1 mRNA expression level and (B) western blot analysis of Notch1 protein expression level in the RCC cell lines 48 h after transfection with miR-34a mimics compared with the negative control. Data are expressed as the mean ± SD. ^*^P<0.05 and ^**^P<0.001, compared with the negative control. All the experiments were performed three times independently. miR-34a, microRNA-34a; RCC, renal cell carcinoma; GAPDH, glyceraldehyde-3-phosphate-dehydrogenase.

Among all the putative and reported genes in the current study, we found that miR-34a significantly reduced the mRNA expression of Notch1 in the 786-O and Caki-1 RCC cell lines. Thus, our study focused on Notch1 and investigated whether the forced expression of miR-34a could downregulate the expression of Notch1 in RCC. The RCC cell lines were transfected with miR-34a mimics and the negative control; after 48 h, the cells transfected with miR-34a mimics displayed decreased Notch1 protein and mRNA levels in comparison to those transfected with the negative control (Fig. 4A and B).

## Discussion

A number of studies have reported a relatively low expression level of miR-34a in human tumors and cancer cell lines (12,18). In accordance with these observations, qPCR examination revealed that the expression of miR-34a was reduced in eight of the 10 (80%) human RCC tissues compared with the adjacent normal tissues (Fig. 1), which suggested that dysregulation of miR-34a may be involved in the development of human RCC. However, a limitation of the present study was the small number of tissue samples. Our study measured the expression of miR-34a in RCC tissue to investigate whether miR-34a is involved in the development of RCC; although, the main purpose of the study was to explore the biological function and molecular mechanism of miR-34a in RCC cell lines. The decreased expression of miR-34a in tumors may be correlated with the loss of chromosome 1p36, p53 mutations and CpG methylation of the miR-34a promoters in cancer. The 1p36 genomic region is frequently deleted in a number of tumors (25) and miR-34a was reported to be located within chromosome 1p36 (12,26). Furthermore, previous studies have reported that miR-34a is the target of p53 tumor suppressor gene, underlying the downregulation of miR-34a in tumors (6,9), and p53 mutation is involved in the development of RCC (27). In addition, miR-34a was inactivated by aberrant CpG methylation in various types of cancer, including RCC (28). Thus, the downregulation of miR-34a may be a common event involved in the tumorigenesis of RCC.

Our study focused on the antiproliferative effects of miR-34a in RCC. Forced expression of miR-34a was observed to reduce the proliferation rate of the RCC cell lines (Fig. 2A and B) compared with the negative control. In addition, Notch signaling plays important roles in the carcinogenesis of RCC. Xu *et al* (29) showed that Notch1 functions as an oncogene, promoting the growth of RCC. It has also been reported that Notch1 is associated with the metastasis of RCC (22,23). Previous studies have shown that Notch1 is the target of miR-34a in a number of tumors (14,15,30). However, to the best of our knowledge, it had not previously been demonstrated that miR-34a downregulates the expression of Notch1 in RCC. Our findings suggested that miR-34a repressed the expression of Notch1 at the protein and mRNA level (Fig. 4). Thus, the suppressive function of miR-34a on the proliferation of RCC cells may be partly due to the regulation of Notch1 and miR-34a may affect the Notch signaling pathway.

Previous studies have reported that miR-34a induces cell cycle arrest in tumors (11,14,15); however, in our study, a significant decrease in the percentage of cells in S phase and a significant G0–G1 arrest was observed in 786-O cells, while no significant decrease in the percentage of cells in S phase, but a significant G0–G1 arrest was identified in Caki-1 cells (Fig. 2C and D). Therefore, there may be alternative mechanisms involved in cell cycle distribution in the Caki-1 RCC cell line, which require further investigation. The control of cell proliferation by miRNAs may be via the regulation of critical cell cycle regulators (31). Our data suggested that miR-34a may, in part, act through additive or synergistic effects of multiple cell-cycle-related proteins, such as CDK6, E2F3 and CCNE2, mediating the dysregulation of cell growth and proliferation in RCCs (Fig. 3).

In conclusion, the expression of miR-34a was lower in RCC tumors compared with the adjacent normal tissues. Several putative target genes of miR-34a were also dysregulated at the mRNA level when the expression of miR-34a was elevated in the RCC cell lines. Our findings suggest that miR-34a could suppress carcinogenesis by modulating the expression of Notch1 at the mRNA and protein level, thus inhibiting cell proliferation and inducing cell cycle arrest at G0 phase in the RCC cell lines. In conclusion, miR-34a expression is associated with the malignant behavior of RCC cells and, therefore, may be a candidate molecular therapeutic target for RCC in the future (32).

## Figures and Tables

**Table I:** Clinicopathological characteristics and miRNA-34a expression in renal cell carcinoma tissues.

No.	Age (years)	Gender	Clinical stage	Pathological grade	Relative expression of miR-34a
1	59	M	II	G2	Downregulated
2	56	M	I	G2	Downregulated
3	71	F	II	G2	Upregulated
4	66	M	I	G2	Downregulated
5	61	F	I	G1	Downregulated
6	64	M	III	G3	Upregulated
7	62	F	II	G2	Downregulated
8	57	M	II	G2	Downregulated
9	57	M	I	G3	Downregulated
10	74	M	II	G2	Downregulated

miR-34a, microRNA-34a; M, male; F, female.

**Table II:** Primer sequences of target genes of microRNA-34a.

Target genes	Forward primer	Reverse primer
SIRT1	5′-TCCTGGACAATTCCAGCCATCTCT-3′	5′-TCCAGCGTGTCTATGTTCTGGGTA-3′
Notch1	5′-TGGAGAAGGGAAGTTGAACGAGCA-3′	5′-CAAATTAATCCGCGTGCGGAAGGT-3′
CDK6	5′-ATTCACTGCCTGGGACACAGTCTT-3′	5′-ACAGGCCACTGTGGTAACTCTCAA-3′
E2F3	5′-TGCAGTGTTGTCCCTTCCTACCTT-3′	5′-GCCTGCAACTGTGCGTTTAGACAA-3′
CCNE2	5′-ATGACACCACCGAAGAGCACTGAA-3′	5′-TGGCTAGGGCAATCAATCACAGCA-3′
GAPDH	5′-TCGACAGTCAGCCGCATCTTCTTT-3′	5′-ACCAAATCCGTTGACTCCGACCTT-3′

SIRT1, silent information regulator 1; CDK6, cyclin-dependent kinase 6; E2F3, E2F transcription factor 3; CCNE2, cyclin E2; GAPDH, glyceraldehyde-3-phosphate-dehydrogenase.

## Abbreviations

RCC: renal cell carcinoma
miR-34a: microRNA-34a
CCK-8: Cell Counting Kit-8
qPCR: quantitative polymerase chain reaction
GAPDH: glyceraldehyde-3-phosphate-dehydrogenase
SIRT1: silent information regulator 1
CDK6: cyclin-dependent kinase 6
CCNE2: cyclin E2
